# Examining disease boundaries: Genetics of myelodysplastic/myeloproliferative neoplasms

**DOI:** 10.1002/jha2.264

**Published:** 2021-07-19

**Authors:** Michael J. Hochman, Bipin N. Savani, Tania Jain

**Affiliations:** ^1^ Division of Hematological Malignancies and Bone Marrow Transplantation Sidney Kimmel Comprehensive Cancer Center Johns Hopkins University Baltimore Maryland USA; ^2^ Division of Hematology and Oncology Vanderbilt University Medical Center Nashville Tennessee USA

**Keywords:** clonal architecture, disease classification, genomic landscapes, MDS/MPN overlap syndromes, myeloid malignancies

## Abstract

Myelodysplastic/myeloproliferative neoplasms (MDS/MPN) are clonal myeloid malignancies that are characterized by dysplasia resulting in cytopenias as well as proliferative features such as thrombocytosis or splenomegaly. Recent studies have better defined the genetics underlying this diverse group of disorders. Trisomy 8, monosomy 7, and loss of Y chromosome are the most common cytogenetic abnormalities seen. Chronic myelomonocytic leukemia (CMML) likely develops from early clones with *TET2* mutations that drive granulomonocytic differentiation. Mutations in *SRSF2* are common and those in the RAS‐MAPK pathway are typically implicated in disease with a proliferative phenotype. Several prognostic systems have incorporated genetic features, with *ASXL1* most consistently demonstrating worse prognosis. Atypical chronic myeloid leukemia (aCML) is most known for granulocytosis with marked dysplasia and often harbors *ASXL1* mutations, but *SETBP1* and *ETNK1* are more specific to this disease. MDS/MPN with ring sideroblasts and thrombocytosis (MDS/MPN‐RS‐T) most commonly involves spliceosome mutations (namely *SF3B1*) and mutations in the JAK‐STAT pathway. Finally, MDS/MPN‐unclassifiable (MDS/MPN‐U) is least characterized but a significant fraction carries mutations in *TP53*. The remaining patients have clinical and/or genetic features similar to the other MDS/MPNs, suggesting there is room to better characterize this entity. Evolution from age‐related clonal hematopoiesis to MDS/MPN likely depends on the order of mutation acquisition and interactions between various biologic factors. Genetics will continue to play a critical role in our understanding of these illnesses and advancing patient care.

## 1 INTRODUCTION

Myelodysplastic/myeloproliferative‐overlap neoplasms (MDS/MPN) are a group of clonal myeloid disorders with characteristics of dysplasia, resulting in dysfunctional hematopoiesis and cytopenias, as well as proliferative features causing increased cell counts, organ infiltration, and constitutional symptoms. They include chronic myelomonocytic leukemia (CMML), atypical chronic myeloid leukemia (aCML), MDS/MPN with ring sideroblasts (RSs) and thrombocytosis (MDS/MPN‐RS‐T), and MDS/MPN‐unclassifiable (MDS/MPN‐U) in adults, while juvenile myelomonocytic leukemia (JMML) is exclusively seen in pediatric populations.

With the advent of next generation sequencing technologies, the genetics and clonal architecture of MDS/MPNs have been increasingly elucidated. Much like the myeloid disorders that is this group's namesake, the mutations underlying the MDS/MPNs affect DNA methylation, chromatin modification, RNA splicing, transcription regulation, cytokine receptors, and proliferative signaling pathways.

Cytogenetically, these syndromes are most defined by the absence of specific chromosomal abnormalities so as to exclude more prevalent diagnoses. For example, all diagnoses must exclude the presence of the *BCR‐ABL* fusion gene seen with t(9;22) [1]. Testing must also be done to ensure there are no rearrangements involving platelet‐derived growth factor A (*PDGFRA*), *PDGFRB*, fibroblast growth factor receptor 1 (*FGFR1*), or pericentriolar material 1 (*PCM1*)‐Janus kinase 2 (*JAK2*) fusions in patients with eosinophilia to exclude this group of myeloid neoplasms [1]. Finally, MDS/MPN‐RS‐T cannot have isolated del(5q), t(3;3)(q21;q26), or inv(3)(q21q26) [1]. Cytogenetic abnormalities are more prevalent in MDS/MPN‐U and aCML than CMML and MDS/MPN‐RS‐T, the most common of which are trisomy 8, monosomy 7/deletion 7q, and loss of Y chromosome [2, 3, 4].

Much of our initial understanding of these diseases was extrapolated from studies of the relatively common MDS and MPNs. Subsequent studies, including both large collaborative and small single center ones, further advanced the knowledge of genetic pathways and their pathological consequences. In this review, we will discuss these findings and their impact on disease prognosis and pathophysiology, then discuss implications for disease characterization.

### 1.1 CMML

The most common of the MDS/MPNs, CMML is defined as a myeloid stem cell neoplasm characterized by presence of greater than 1000 monocytes/μl in the peripheral blood (PB), with monocytes comprising at least 10% of PB white blood cells (WBCs), evidence of dysplasia in at least one lineage, and less than 20% blasts [1]. Monocyte partitioning by flow cytometry may distinguish reactive from classical monocytes and aid in diagnosis [5]. It is often diagnosed incidentally or in the context of cytopenias or antecedent autoimmune illness [6, 7]. Patients may have a dysplastic phenotype characterized by low counts or a proliferative one characterized by leukocytosis (≥13,000 WBC/μl) and hepatosplenomegaly. A notable characteristic observed in vitro is hypersensitivity to granulocyte‐macrophage colony‐stimulating factor (GM‐CSF) [8], prompting study of the anti‐GM‐CSF monoclonal antibody lenzilumab [9].

Most genetic abnormalities in CMML involve mutations of a select few genes involved in DNA methylation, splicing, and proliferative signaling. Ten‐eleven translocation‐2 (*TET2*) is the most commonly mutated gene, seen in 50%–70% of patients, [3, 10, 11] followed by *SRSF2* (∼50%) and additional sex combs‐like 1 gene (*ASXL1*; ∼40%). Biallelic mutation of *TET2* has been frequently recognized [3, 11]. Mutations in the RAS pathway (e.g., *NRAS*, *KRAS*, casitas B‐lineage lymphoma [*CBL*], *PTPN11*) are also frequently observed, in up to 40% of patients [12]. Karyotype abnormalities exist in less than a quarter of CMML patients [3,4] and typically involve chromosomes 7 and 8. Patients with therapy‐related CMML may have more frequent cytogenetic abnormalities but carry a similar mutation burden [13].

*TET2* plays an important role in DNA methylation balance by performing hydroxylation of methylated cytosine residues, facilitating demethylation of these nucleotide residues. Mutations cause a loss of function, resulting in aberrant DNA methylation, dysmyelopoiesis, and clonal expansion [14, 15, 16]. One model of CMML pathogenesis suggests that early clonal dominance of *TET2*‐mutant (*TET* ^MT^) clones promotes granulomonocytic differentiation of immature progenitors [17], whereas without this early dominance, additional mutations or other stochastic factors cause evolution toward other phenotypes.

Given the increased presence of mutations associated with age‐related clonal hematopoiesis in CMML [18], notably *TET2* and *ASXL1*, CMML appears to stem from hematopoietic cells that stochastically acquire culprit mutations over time. Ancestral *TET2* mutations are most common [19] and are highly associated with biallelic *TET2* mutation and *TET2*‐*SRSF2* co‐mutation [3]. Additionally, *ASXL1* has been found to be a common ancestral event [3, 19]. The sequence of driver mutation acquisition and gene interactions likely skews disease toward either a more proliferative or dysplastic phenotype [20] with RAS pathway mutations responsible for the former [12] and *SF3B1* occasionally associated with the latter [21].

Genetic features have been incorporated into multiple prognostic models. A large Spanish registry categorized cytogenetic risk based on overall survival (OS) and progression to acute myeloid leukemia (AML): low risk (normal karyotype or isolated loss of Y chromosome), high risk (trisomy 8, abnormalities of chromosome 7, or complex karyotype [CK]), and intermediate (all other abnormalities) [22]. These risk groups were later incorporated into the CMML‐specific prognostic scoring system (CPSS) [23]. Published the same year, the *Groupe Français des Myélodysplasies* prognostic score included *ASXL1* nonsense and frameshift mutations because they conferred poor prognosis in multivariate analysis [10], confirmed later in a larger study [24]. Subsequently, *ASXL1*, *NRAS*, *RUNX1*, and SET‐binding protein‐1 (*SETBP1)* were independently linked to worse OS and increased risk of leukemic transformation. These mutations were integrated with the established cytogenetic risk categories into a CMML‐specific genetic score, which was incorporated into the clinical/molecular CPSS (CPSS‐Mol) [25].

Although a smaller study found that patients with *TET2* ^MT^ CMML‐1 had adverse prognosis [11], this has not been consistently observed. In one large, multi‐institutional study, not only was *TET2* ^MT^ disease associated with a significant survival advantage, but truncating or multiple *TET2* mutations were particularly advantageous [26]. Additionally, the impact of *ASXL1* was found to be ameliorated by co‐mutation with *TET2* in this study [26]. Finally, another study found mutations in *STAG2* and *U2AF1* correlated with worse survival [3].

There have been multiple attempts to use genetic and epigenetic signatures to predict response to therapy. While somatic mutations did not predict response to hypomethylating agent (HMA) therapy in one international study, methylation profiling of mostly nonpromoter regions distinguished responders from non‐responders [27]. Gene profiling of the responders found that expression of cell cycle‐related genes were upregulated. In other evaluations, mutations in *RUNX1* and *CBL* predicted worse OS in patients undergoing HMA therapy, but *TET2* ^MT^and *ASXL1*‐wildtype (*ASXL1* ^WT^) genotypes had better outcomes [28, 29]. Interestingly, HMA therapy does not appear to alter disease mutation burden or be cytotoxic to cancer cells but instead alters gene expression through epigenetic changes [30]. Finally, genetics impact response to hematopoietic stem cell transplantation: patients with high overall mutation burden (>10 mutations) were found to be at higher risk of relapse [31]. In a recently developed prognostic model, *ASXL1* and *NRAS* mutations independently worsened survival in CMML patients post‐transplant [32].

### 1.2 JMML

Unlike CMML, JMML is a disease of infancy and early childhood characterized by organ infiltration of malignant cells resulting in lymphadenopathy, hepatosplenomegaly, cutaneous lesions, as well as constitutional symptoms and infectious sequelae. Its canonical feature is hypersensitivity to GM‐CSF [33], as seen in CMML, thought to be related to its nature as a RASopathy. Indeed, most cases arise from somatic or germline mutations in genes involving the RAS‐MAPK pathway: *PTPN11*, *N/KRAS*, *NF1*, *or CBL* are seen in about 90% of cases [34]. Mutations involving other signal transduction pathways, splicing, transcription, and polycomb repressive complex 2 have also been described [35]. Two‐thirds of patients will have normal karyotype, but the remaining will have monosomy 7 (26%) and 10% with other aberrancies [36].

### 1.3 aCML

This entity is characterized by neutrophilia with severe dysgranulopoiesis, along with bone marrow findings of hypercellularity and granulocytic dysplasia [37]. Perhaps due to significant clinical, morphologic, and genetic overlap with the MPN chronic neutrophilic leukemia (CNL) [38], WHO guidelines advise that granulocyte precursors (promyelocytes, myelocytes, and metamyelocytes) should comprise at least 10% of PB WBCs [1]. To exclude CML and CMML, there must be no or minimal absolute basophilia or monocytosis as well as no evidence of *BCR‐ABL* [1]. This disease is considered relatively aggressive, with a median OS of about 11–14 months [37,39,40] and a known propensity for leukemic transformation.

aCML is most specifically associated with mutations in *SETBP1* and ethanolamine kinase 1 (*ETNK1)*, which typically are found in 23%–38% and 3%–9% of patients, respectively [3, 4, 41, 42, 43]. As *SETBP1* has a lower prevalence in other MDS/MPN except CMML or CNL, and *ETNK1* is only otherwise seen in CMML and systemic mastocytosis [43], these genetic markers can be useful to distinguish aCML from other myeloid neoplasms. Less specific but far more prevalent is *ASXL1*, the most frequently mutated gene (60%–90%) [3, 4]. *SETBP1* was found to be equally codominant or secondary to *ASXL1* in aCML as *ASXL1* mutations were found in a high percentage (>90%) of ancestral clones [3]. Other common mutations in aCML are *TET2* (43%), *SRSF2* (34%), *NRAS* (31%) [4], *EZH2* (>30%) [3], *RUNX1* (20%) [3, 4], and *CBL* (8%) [44]. Although mutations in the granulocyte‐colony stimulating factor 3 receptor (*CSF3R*) were previously thought to be closely linked to both aCML and CNL [45], it is now recognized to be more common in CNL [1, 46, 47]. About 40% of aCML patients have an abnormal karyotype [3, 4, 40].

Evaluation of the most aCML‐specific mutations has yielded insight into the pathophysiology of this disease. For instance, *SETBP1*, found on the long arm of chromosome 18, plays a role in cell proliferation by producing a nuclear protein that binds SET and inhibits the tumor suppressor gene *PP2A* [42, 48]. Overexpression of this gene is associated with poor outcome in AML, and it may represent a unique mechanism in leukemogenesis [48, 49].

In 2015, heterozygous mutation of *ETNK1* was recognized as an abnormality largely unique to aCML [43]. As this enzyme is the first in a specific phospholipid biosynthesis pathway, its loss of catalytic function may impact cell membrane architecture, cytokinesis during cell division [50], and optimal respiratory chain performance in the mitochondrial inner membrane [51]. In fact, a recent study found that the metabolic product of *ETNK1* attenuates mitochondrial activity, with loss of function driving increased production of reactive oxygen species (ROS) and mutagenesis. In vitro experiments suggested a normal phenotype can be restored by administering the underproduced enzyme product or the antibiotic tigecycline [52], which is known to inhibit synthesis of mitochondrial proteins that enable ROS production.

Despite evidence that *SETBP1* confers worse prognosis in myeloid malignancies in general [53, 54] and aCML specifically [42], its impact has not been found deleterious in all studies [3, 40]. The frequent coexistence of mutations in *ASXL1* and *CBL* with *SETBP1* suggests an association with worse outcomes; [41, 42] *CBL* mutation alone suggests more aggressive disease [44]. Notably, *TET2* mutation is independently associated with worse OS in aCML [40], although may be mutually exclusive with mutations in *SETBP1* [41]. Finally, *SRSF2* mutations may correlate with better OS while those in *RUNX1*, *NRAS*, and *CUX1* suggest poor outcomes [3].

### 1.4 MDS/MPN‐RS‐T

Only upgraded from a provisional entity in the 2016 WHO classification of myeloid neoplasms [1], MDS/MPN‐RS‐T is essentially the epitome of overlap between these two disease spectrums. As its name suggests, greater than 15% RSs are found on marrow iron stain [1]. These erythroid precursors have a perinuclear ring of blue granules on Prussian blue stain, reflective of mitochondrial iron overload resulting in dyserythropoiesis [55]. Proliferative mutations drive elevated platelet counts, arterial and venous thrombosis [56, 57], vasomotor symptoms, and bleeding from acquired von Willebrand syndrome [58]. Relative to the other MDS/MPNs, prognosis is better (median OS 76 months) [57].

Similar to its phenotype, the genotype of this disease is a melding of stereotypical dysplastic and proliferative genetic aberrations. Founder mutations are frequently in genes involved in RNA splicing: *SF3B1* is found in about 90% of patients [4], and *SF3B1* ^WT^ patients typically harbor other spliceosome mutations [59]. *SF3B1* encodes subunit 1 of the splicing factor 3b complex and may cause RS formation via downregulation of key mitochondrial gene networks [60]. Curiously, in vitro experiments showed hotspot *SF3B1* mutations induce cellular metabolic changes by aberrant mRNA splicing, causing defects in the synthesis of the non‐essential amino acid serine and thereby inducing a vulnerability to serine deprivation [61]. Proliferative features are attributed to mutations involving the JAK‐STAT pathway, with about half of patients having *JAK2* V617F(59) or less frequently (<10%) mutations in *CALR* or *MPL* [1]. One model suggests that disease develops from an *SF3B1* ^MT^ clone with RSs, which then develops thrombocytosis following somatic mutation in the JAK‐STAT pathway [62].

Mutation in DNA (cytosine‐5)‐methyltransferase 3 alpha (*DNMT3A)*, a gene involved in epigenetic regulation via DNA methylation, is associated with both MDS/MPN‐RS‐T and myelodysplasia with RSs (MDS‐RS) [63]. Palomo et al found that *DNMT3A* was always the founder mutation in MDS/MPN‐RS‐T patients who had it, whereas *ASXL1* and *TET2* never originated prior to *SF3B1* [3]. Perhaps *DNMT3A* mutation is well‐suited to promoting a RS phenotype; patients with a *DNMT3A* ^MT^/*SF3B1* ^MT^ genotype were found to have a higher percentage of RS than *DNMT3A* ^WT^/*SF3B1* ^MT^ cases [59]. Although *JAK2* mutation is classically associated with thrombosis in MPNs, there is evidence that *SF3B1* also increases the thrombosis risk in both MDS‐RS and MDS‐RS‐T [56, 63]. Unlike CMML, the RAS‐MAPK pathway is not commonly affected in MDS/MPN‐RS‐T [3].

Like in CMML, cytogenetic abnormalities are uncommon and found in about only 10%–20% of patients [3, 4, 55]. Abnormal karyotype and mutations in *ASXL1* and *SETBP1* are associated with worse OS in this population [3, 64]. These mutations were found in 29% and 13% of patients in one cohort, respectively [64]. *EZH2* also correlates with worse OS [3].

### 1.5 MDS/MPN‐U

The least well‐defined of the chronic myeloid neoplasms, MDS/MPN‐U is defined as a malignancy with dysplastic features in at least one cell lineage, prominent myeloproliferative features (either platelet count ≥450,000/μl or WBC ≥13,000/μl), and <20% blasts in blood and bone marrow. Splenomegaly may be seen. Patients must not have a history of either MDS or MPN nor recent cytotoxic growth factor therapy. The median OS is 12.4 months with thrombocytosis being an independent positive prognostic factor [65].

The genetic abnormalities found in MDS/MPN‐U are broad. *ASXL1* is the most frequent mutation, occurring in about 30%–50% of patients. Others include *TET2*, *JAK2*, *EZH2*, *SRSF2*, *NRAS*, *SETBP1*, *RUNX1*, *STAG2*, *U2AF1*, and *TP53* [2, 3, 4, 66]. Diploid cytogenetics are seen in 49%–71% of patients [2–4,65], and about 12% of patients have CK [2, 3, 65]. *CBL* and *TP53* are independent risk factors for worse prognosis [2], whereas *ASXL1*, *EZH2*, and *STAG2* mutations are associated with worse survival [3].

In a large (*n* = 106) study of MDS/MPN‐U clonal architecture, unclassifiable cases actually segregated into subgroups that fit the other MDS/MPNs based on genetic and clinical features [3]: 17% had a CMML‐like signature, 33% were aCML‐like, and 11% had MDS/MPN‐RS‐T‐like disease. The CMML‐like disease, for instance, included patients with either biallelic *TET2* mutations, *TET2* and *SRSF2* co‐mutation, or *RUNX1* and *SRSF2* co‐mutation; survival curves were similar between this group and WHO‐classified CMML patients. Patients with mono‐ or biallelic mutations in *TP53* segregated into a fourth group (13%), characterized by worse anemia, higher bone marrow blast percentage, and worse prognosis [3]. Finally, the remaining 26% of patients more frequently had mutations in *JAK2*, *U2AF1*, and *ASXL1* and were more likely to have thrombocytosis. Survival in these “other” patients trended worse than CMML‐like patients but was better than aCML‐like patients.

## 2 DISCUSSION

It was not until 2001 that the WHO re‐classified CMML from a subset of MDS to the newly created MDS/MPN category [67]. Since then, there have been significant advancements in understanding this group of neoplasms (Table 1) and an increase in clinical trials targeting CMML patients in particular. There remains a need to better characterize MDS/MPNs by genetic features—to more accurately understand the natural history of the disease and ensure patient eligibility for clinical trials and FDA‐approved treatment options [68]. Additionally, genetics plays a critical role in disease prognostication; for instance, the worse outcomes seen among men with MDS/MPN as compared to women may be explained by gender‐related differences in somatic mutation profiles [69]. Our review highlights the advances in our understanding of the genetics of these diseases.

**TABLE 1:** Overview of diagnostic, clinical, and genetic features of the MDS/MPN overlap syndromes. MDS/MPNs all must have <20% blasts in PB and BM, with the RS‐T subtype only allowing for <1% PB and <5% BM blasts. No evidence of the *BCR‐ABL1* fusion gene, *PDGFR*, *FGFR1*, and *PCM1‐JAK2* is permitted. No history of cytotoxic or growth factor therapy is permitted in the RS‐T or U subtypes

Disease	2016 WHO diagnostic criteria	Clinical features	Cytogenetics	Molecular genetics	Other
**CMML**	‐ PB monocytosis > 1000/ul with monocytes ≥10% of WBCs ‐ One of the below 4: ‐ ≥1 myeloid lineage with dysplasia ‐ Acquired clonal genetic abnormality present ‐ Monocytosis persisting ≥3 months with other causes excluded ‐ Classical (CD14+/CD16‐) monocyte immunophenotype by flow cytometry	‐ May be associated with antecedent autoimmune disease ‐ Organomegaly, effusions are proliferative features seen ‐ PB WBC defines proliferative (≥13,000/μl) versus dysplastic (<13,000/μl) types	‐ Majority (∼70%) patients have NK ‐ Abnormal karyotype more common in therapy‐related disease ‐ Most common abnormalities: +8, monosomy 7, −Y, +21, del(20q) ‐ Low risk abnormalities: NK, −Y ‐ High risk abnormalities: +8, chromosome 7 abnormalities, CK	‐ Most common: *TET2* followed by *SRSF2*, *ASXL1* ‐ RAS pathway mutations also commonly seen in proliferative disease ‐ High risk mutations: ‐ *ASXL1*, *NRAS*, *RUNX1*, and *SETBP1* ‐ *ASXL1* and *NRAS* linked to worse HCT outcomes	‐ PB WBC and blast % (in PB and BM) affect prognosis ‐ Methylation profiling may distinguish HMA responders from non‐responders ‐ Oligomonocytic CMML (PB monocytosis < 1000/μl) recognized but not defined ‐ Ruxolitinib under study as novel therapy (NCT03722407)
**JMML**	‐ PB monocyte count > 1000/ul, splenomegaly, absence of *BCR‐ABL1* ‐ One of the below: ‐ Somatic mutation in *PTPN11*, *KRAS*, or *NRAS* ‐ NF1 diagnosis or *NF1* mutation ‐ Germ line *CBL* mutation and LOH of CBL ‐ Chromosomal abnormality such as monosomy 7 ‐ Two of the following: HbF increased for age, myeloid or erythroid precursors on PB smear, GM‐CSF hypersensitivity on colony assay, hyperphosphorylation of STAT5	‐ Disease of infancy and early childhood ‐ Clinical findings include lymphadenopathy, hepatosplenomegaly, cutaneous lesions, constitutional symptoms	‐ Majority (∼75%) of patients have NK ‐ Monosomy 7 most common abnormality	‐ RAS‐MAPK pathway mutations common (85% of patients): *PTPN11*, *N/KRAS*, *NF1*, or *CBL* ‐ *PTPN11* most common ‐ Coexisting RAS‐MAPK mutations found in about 1/10 of patients ‐ *SETBP1*, *JAK3* rarely found	‐ Associated with congenital diseases such as Noonan syndrome, neurofibromatosis type 1, and a germline syndrome associated with the *CBL* mutation
**aCML**	‐ PB leukocytosis with increased neutrophils and granulocytic precursors with ≥10% WBCs ‐ Basophils are <2% WBCs ‐ Monocytes are <10% WBCs ‐ Not meeting criteria for other MPN ‐ Hypercellular BM with granulocytic dysplasia	‐ Relatively aggressive: Median overall survival 11–14 months ‐ Clinical findings include leukocytosis and neutrophilia ‐ High rates of leukemic transformation	‐ Majority (∼60%) of patients have NK ‐ Most common abnormalities: +8, −7, CK	‐ Most common: *ASXL1*, *TET2*, *SRSF2*, *NRAS*, *EZH2*, *RUNX1*, *CBL* ‐ Most specific: *SETBP1*, *ETNK1* ‐ *CSF3R* more common in CNL than aCML ‐ High risk mutations: ‐ *SETBP1*, *ASXL1*, *CBL*, *TET2*	‐ Normal role of *SETBP1* involves inhibition of tumor suppressor gene *PP2A* ‐ *ETNK1* involved in phospholipid biosynthesis has downstream impact on mitochondrial function
**MDS/MPN‐RS‐T**	‐ Anemia with erythroid lineage dysplasia ‐ ≥15% BM RS ‐ Persistent thrombocytosis ≥450K ‐ Presence of *SF3B1* mutation ‐ If not present, patient must not have history of cytotoxic or growth factor therapy ‐ No t(3;3), inv(3), del(5q) ‐ No history of myeloid clonal neoplasm (aside from MDS‐RS)	‐ Relatively better prognosis ‐ Clinical findings include arterial and venous thrombosis, vasomotor symptoms, bleeding from aVWS	‐ Majority (∼90%) have NK ‐ High risk abnormalities: any cytogenetic abnormality	‐ Most common: ‐ Splicing mutations, namely *SF3B1* ‐ JAK‐STAT pathway mutations: *JAK2*, *CALR*, *MPL* ‐ *ASXL1*, *DNMT3A*, *TET2* ‐ High risk mutations: *ASXL1*, *SETBP1*	‐ *SF3B1* specifically associated with increased thrombosis risk
**MDS/MPN‐U**	‐ Dysplastic features in at least one blood cell type ‐ Prominent myeloproliferative feature(s) ‐ No isolated del(5q) ‐ Not fitting any other category	‐ Median OS 12.4 months ‐ Outcomes more favorable in patients < 60 years old, with thrombocytosis, without circulating blasts, <5% BM blasts	‐ Most patients have NK (49%–71%) ‐ Most common abnormalities: trisomy 8, −7, CK	‐ Most common: *ASXL1*, *TET2*, *JAK2*, *EZH2*, *SRSF2*, *NRAS*, *SETBP1*, *RUNX1*, *STAG2*, *U2AF1*, *TP53* ‐ High risk: presence of one mutation, *TP53*	‐ Studies have identified subgroups based on genetic/clinical features resembling other MDS/MPNs

Abbreviations: aCML, atypical chronic myeloid leukemia; aVWS, acquired von Willebrand syndrome; BM, bone marrow; CK, complex karyotype; CMML, chronic myelomonocytic leukemia; CNL, chronic neutrophilic leukemia; HCT, hematopoietic cell transplantation; HMA, hypomethylating agents; JMML, juvenile myelomonocytic leukemia; LOH, loss of heterozygosity; MDS, myelodysplasia; MPN, myeloproliferative neoplasm; NK, normal karyotype; OS, overall survival; PB, peripheral blood; RS‐T, ring sideroblasts with thrombocytosis; U, unclassifiable.

As observers of natural phenomena, our inclination is to categorize disease based on features such as cell count abnormalities, morphology, and genetic signatures. If the cutoffs for these characteristics are not specific enough, then disease classifications become less meaningful. However, if the prescribed cutoffs are too restrictive, meaningful data about cases that fall outside rigid parameters may be lost into an exclusionary “wastebasket” category. Thoughtfully liberalizing criteria can notably help encompass cases that may be at a more immature stage, as done in 2016: The criteria for MDS‐RS were adjusted from requiring ≥15% RS on marrow exam to needing ≥5% RS in the presence of *SF3B1* mutation [1]. Conceivably, these cases represent disease that may eventually progress to ≥15% RS or simply have prognoses that are not meaningfully different than those defined by the pre‐2016 classifications.

**FIGURE 1:**
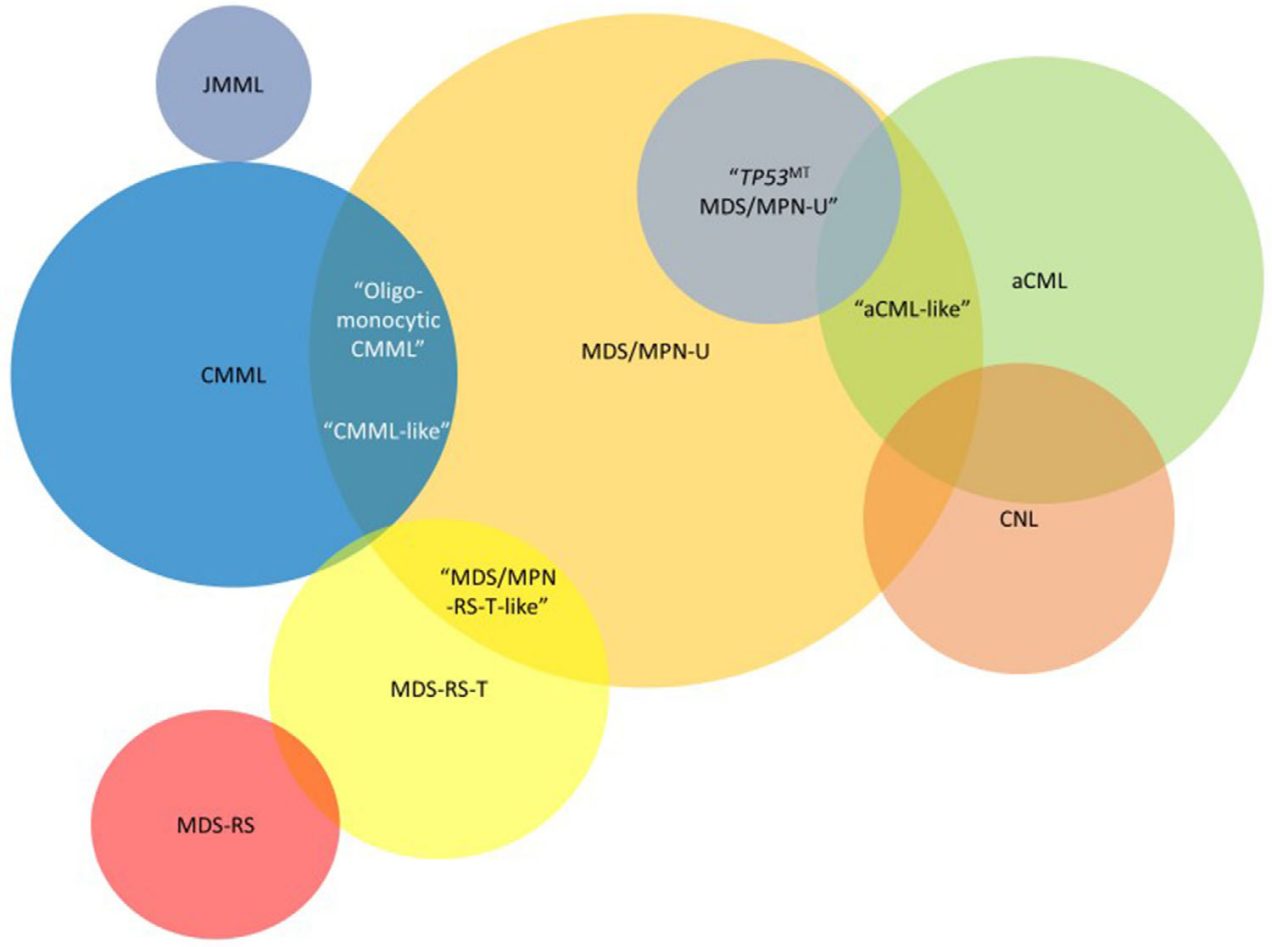
Diagram depicting the MDS/MPN as defined by genetic and clinical features, which may overlap themselves due to genetic heterogeneity or early‐stage disease. Within these are several entities (in quotation marks) that represent diseases with similar genetics and clinical characteristics, but not meeting WHO criteria Abbreviations: aCML, atypical chronic myeloid leukemia; CMML, chronic myelomonocytic leukemia; CNL, chronic neutrophilic leukemia; JMML, juvenile myelomonocytic leukemia; MDS, myelodysplastic syndrome; MPN, myeloproliferative neoplasm; MDS‐RS, myelodysplasia with ring sideroblasts; MDS/MPN‐RS‐T, ring sideroblast with thrombocytosis; MDS/MPN‐U, unclassifiable.

Evidence suggests that such disease exists in the MDS/MPNs and is frequently labeled under alternative categories (Figure 1). For example, “oligomonocytic” CMML shares genetic and clinical features with CMML but does not meet the WHO criteria due to absolute monocytosis < 1000/μl [70, 71], likely representing early‐phase dysplastic‐type CMML. A similar example is found within a group of MDS/MPN‐U patients with ≥15% RS on bone marrow and enriched for *JAK2* and *SF3B1* mutations. Although they did not meet criteria for MDS/MPN‐RS‐T due to lack of thrombocytosis or other features, they had prognoses similar to an independent cohort of patients with MDS/MPN‐RS‐T [2].

Our current classifications likely do not allow for enough nuance, too. Although there is evidence that the less well‐defined chronic myeloid malignancies are distinct entities based on cytogenetic risk stratification and leukemia free survival [39], a more recent study found significant heterogeneity among patients with CNL, aCML, MPN‐U, MDS/MPN‐U, and CMML [15]. Based on whole exome and RNA sequencing, researchers identified at least 15 groups of patients with different combinatorial mutations patterns, suggesting these diseases represent a continuum as opposed to distinct pathologies. Perhaps disease classification for these more genetically diverse entities should be more granular by specifying aberrations in their names, as done with some AML subtypes [1].

## 3 SUMMARY AND FUTURE DIRECTIONS

The MDS/MPNs are rare and heterogeneous, making systematic study more difficult relative to other myeloid malignancies. Advances in gene sequencing technology along with large multi‐institution collaborations have shed light on these illnesses: We now know that most have signaling gene mutations responsible for proliferative phenotypes, and many have gene mutations affecting epigenetic regulation, causing dysplastic features. These syndromes likely often evolve out of preexisting clones that mature into neoplasms with phenotypes based on the order of mutation acquisition and interaction between epigenetic and genetic factors. Genetic studies will likely aid in revising WHO criteria so as to more holistically classify the overlap syndromes.

## CONFLICT OF INTEREST

Tania Jain serves as a consultant for Targeted Healthcare Communications and has served on the advisory board for CareDx and Bristol Myers Squibb. The authors Michael J. Hochman and Bipin Savani have no disclosures.

## AUTHOR CONTRIBUTIONS

Michael J. Hochman did the literature review and wrote the manuscript. Bipin Savani did the literature search and reviewed the manuscript. Tania Jain did the literature search, reviewed the manuscript and supervised the project. All authors approve the final draft of the manuscript.
